# Construction of cuproptosis-related gene signature to predict the prognosis and immunotherapy efficacy of patients with bladder cancer through bioinformatics analysis and experimental validation

**DOI:** 10.3389/fgene.2022.1074981

**Published:** 2022-11-23

**Authors:** Zijian Liu, Fubin Zhu, Pu Zhang, Bei Qian, Weihui Liu, Yajun Xiao, Nianyong Chen, Qingliu He, Jianghong Xiao

**Affiliations:** ^1^ Department of Head and Neck Oncology, Cancer Center West China Hospital, Sichuan University, Chengdu, China; ^2^ Department of Radiation Oncology, Cancer Center West China Hospital, Sichuan University, Wuhan, China; ^3^ Department of Urology Surgery, Union Hospital, Tongji Medical College, Huazhong University of Science and Technology, Wuhan, China; ^4^ Department of Thyroid and Breast Surgery, Union Hospital, Tongji Medical College, Huazhong University of Science and Technology, Wuhan, China; ^5^ Department of Urology, The Second Affiliated Hospital of Fujian Medical University, Quanzhou, China

**Keywords:** cuproptosis, tumor microenvironment, bladder cancer, immunotherapy, prognosis

## Abstract

**Background:** A new form of cell death, copper-dependent cell death (termed cuproptosis), was illustrated in a recent scientific study. However, the biological function or prognostic value of cuproptosis regulators in bladder cancer (BLCA) remains unknown.

**Materials and Methods:** Sequencing data obtained from BLCA samples in TCGA and GEO databases were preprocessed for analysis. Biological function and immune cell infiltration levels evaluated by gene set variation analysis (GSVA) were employed to calculate enrichment scores. Iteration least absolute shrinkage and selection operator (LASSO) and COX regression model were employed to select feature genes and construct a novel cuproptosis-related (CR) score signature. The genomics of drug sensitivity in cancer (GDSC) and tumor immune dysfunction and exclusion (TIDE) analysis were used to predict the chemotherapy and immunotherapy efficacy for BLCA patients. The relative expression of the genes involved in the signature was also verified by real-time quantitative PCR (qRT-PCR) in cell lines and tissues.

**Results:** Expression abundance and the prognostic value of cuproptosis regulators proved that cuproptosis might play a vital part in the carcinogenesis of BLCA. GSVA revealed that cuproptosis regulators might be associated with metabolism and metastasis-related pathways such as TGF-β, protein secretion, oxidative Phosphorylation, MYC targets, MTORC1, and adipogenesis pathways. CR scores could predict the prognosis and evaluate the chemotherapy and immunotherapy efficacies of BLCA. CR scores were positively correlated with EMT, MYC, MTORC1, HEDGEHOG, and E2F signaling pathways; meanwhile, they were negatively correlated with several immune cell infiltration levels such as CD8^+^ T cells, γδT cells, and activated dendritic cells. Several GEO datasets were used to validate the power of prognostic prediction, and a nomogram was also established for clinical use. The expressions of DDX10, RBM34, and RPL17 were significantly higher in BLCA cell lines and tissues in comparison with those in the corresponding normal controls.

**Conclusion:** Cuproptosis might play an essential role in the progression of BLCA. CR scores could be helpful in the investigation of prognostic prediction and therapeutic efficacy and could make contributions to further studies in BLCA.

## Introduction

With an estimated 549,000 new cases and 200,000 deaths in 2018, as a urinary tract malignancy, bladder cancer is the 10th most common form of cancer globally (Sung et al., 2021). According to the pathological diagnosis, bladder cancer can be divided into two subtypes. One is a non-muscle-invasive subtype (NMIBC), and the other one is an invasive muscle subtype (MIBC) (Prout et al., 1992). While the 5-year survival rate of patients with NMIBC is more than 90%, the 5-year survival rate of patients with MIBC is lower than 70% (Prout et al., 1992). Therefore, much manpower and material resources are inputted, and we also witnessed great advancement in the diagnosis and therapy of bladder cancer (Soloway, 2013). However, the effect of surgery and chemotherapy is still unsatisfactory. Hence, the deeper mechanism of occurrence and progress of bladder cancer is to be urgently found.

As we all know, metal ions are essential for vital movements; however, excessive intracellular accumulation of metal ions could lead to various types of cell deaths, such as ferroptosis induced by iron, etc. (Dixon et al., 2012). Cuproptosis, a novel form of cell death induced by copper ion, has been proven to play an essential role in developing several types of cancers. (Wang et al., 2022). Several genes, such as CDKN2A, FDX1, DLAT, DLD, GLS, LIAS, LIPT1, and MTF1, have been proven to be involved in the process of cuproptosis (Tsvetkov et al., 2022), and their potential mutual interaction mechanism has been explored in clear-cell renal-cell carcinoma, melanoma, colorectal cancer, lung adenocarcinoma, and gastric cancer (Ji et al., 2022; Lv et al., 2022). Although their role in the process of cuproptosis has been reported in recent months, the possible mechanism of these cuproptosis regulators in bladder cancer has never been researched yet. Several studies have reported that ferroptosis regulators could construct prognostic signatures to predict the prognosis or therapeutic efficacy in bladder cancer (Sun et al., 2021; Yan et al., 2021; Yang et al., 2021). Similarly, multiple gene signatures, such as lncRNAs (Wu et al., 2020; Zhou et al., 2021), RNA methylation (Li et al., 2021; Zheng et al., 2021), and immune-related genes (Wang et al., 2020; Jin et al., 2021), were used to emphasize the underlying research value of the genes involved in the signatures. Most studies have only used these gene signatures to construct prognostic models, but few studies have explored the value of the genes involved in the signatures themselves for studying the disease. Nevertheless, systematical analyses of cuproptosis regulators and their related prognostic signatures have never been conducted in BLCA.

Our research aimed to comprehensively analyze the underlying mechanisms between the expression of cuproptosis regulators and enrichments of functional biological pathways. Based on the iteration LASSO and COX regression models, a novel cuproptosis related (CR) signature containing ten cuproptosis-related genes was constructed to predict the overall survival and immunotherapy efficacy of BLCA, and a nomogram was also established for clinical use. Moreover, we illustrated the validity of the prognostic signature from a biological perspective and tested the predictive accuracy in several validation datasets. We surprisingly found that the CR scores could also predict the response of chemotherapy and immunotherapy efficacies in bladder cancer. Eventually, we conducted qRT-PCR in several BLCA cell lines and tissues to verify the expression levels of genes involved in CR signature. These results may provide novel insights to the treatment of bladder cancer.

## Materials and methods

### Dataset source and data preprocessing

The fragments per kilobase per million values and clinical data of BLCA in the Cancer Genome Atlas (TCGA) database were downloaded from the UCSC XENA database (https://xenabrowser.net/datapages/) (Goldman et al., 2020). The GISTIC copy number of BLCA derived from focal copy number estimates was also downloaded from XENA. Microarray profiles were downloaded as the raw “CEL” files from the Gene Expression Omnibus (GEO) database (https://www.ncbi.nlm.nih.gov/geo/), and seven BLCA datasets (GSE13507, GSE32548, GSE32894, GSE48075, GSE48276, GSE69795, and GSE70691) were used in this study (Supplementary Table S1). The “ComBat” algorithm was applied to reduce the likelihood of batch effects from non-biological technical biases between different datasets (Johnson et al., 2007). The immunotherapeutic cohort of metastatic urothelial cancer patients treated with an anti-PD-L1 antibody atezolizumab (IMvigor210 cohort) was used as the validation cohort, and the expression data and detailed clinical annotations were obtained from http://research-pub.Gene.com/imvigor210corebiologies based on Creative Commons 3.0 License. The combined expression profiles of genes in GTEx and TCGA were downloaded from an analysis platform Sangerbox (http://sangerbox.com/home.html) (Shen et al., 2022). Furthermore, the IHC images of genes involved in the CR signature were downloaded from the Human Protein Atlas (https://www.proteinatlas.org).

### Gene set variation analysis

The enrichment scores of curated pathways and infiltration immune cells were quantified by R package “GSVA,” a method used to estimate the variation of gene set enrichment in a single sample (Hänzelmann et al., 2013). The gene set of “c5.all.v6.2. Symbol” was downloaded from the MSigDB database (https://www.gsea-msigdb.org), and a set of immune cell markers containing 24 types of immune cells was obtained from a published article (Supplementary Table S2) (Bindea et al., 2013).

## Estimation of infiltration levels of immune cells

Infiltration levels for distinct immune cells in BLCA were quantified by using the “CIBERSORT” R package (Newman et al., 2015) and employing the LM22 signature and 1,000 permutations. The Estimation of STromal and Immune cells in MAlignant Tumors using Expression data (ESTIMATE) algorithm was applied to the normalized expression matrix for estimating the stromal and immune scores for each BLCA sample (Yoshihara et al., 2013).

### Logistic regression model construction

The logistic regression analysis was performed to screen the characteristic variables with survival significance which would appear with higher frequency during the operation of LASSO. Then, the variables with higher frequencies will be selected for subsequent penalized multivariate Cox proportional hazards survival modeling using an algorithm for variable selection based on L1-penalized estimation. Cross-validation was selected *via* the learning series, and a penalty parameter, λ1, was inflicted upon the gene expression levels during the modeling process. Subsequently, CR was calculated by a combined signature to predict the overall survival of BLCA. Furthermore, a nomogram of the ten cuproptosis regulators was built through the R package “rms” to indicate the OS probability and death odds. The predictive accuracy of the nomogram was tested through a calibration plot.

### Prediction of immunotherapy and chemotherapy response

Tumor Immune Dysfunction and Exclusion (TIDE) database (http://tide.dfci.harvard.edu/) was used to predict the response to immunotherapy in patients (Jiang et al., 2018). The TIDE value was calculated and used to assess the probability of immunotherapy response, and the cutoff of the TIDE value defaulted as 0. The chemotherapeutic response for each sample was predicted according to the largest public pharmacogenomics database, the Genomics of Drug Sensitivity in Cancer (GDSC) (https://www.cancerrxgene.org/). We used the R package to implement “pRRophetic”, the prediction process, where the samples” half-maximal inhibitory concentration (IC50) was evaluated following the instructions (Geeleher et al., 2014).

### Calculation of stemness index and ferroptosis index

To assess the stemness of cancer cells, the gene expression-based stemness index (mRNAsi) was calculated with the instruction of a one-class logistic regression algorithm for each BLCA sample (Malta et al., 2018). To represent the ferroptosis level, a ferroptosis index (FPI) was established based on the expression data of genes in ferroptosis, including positive components and negative components, with instructions published before (Liu et al., 2020).

### Cell culture

The normal uroepithelial cell line SV-HUC-1 and bladder cancer cell lines, including 5,637, UM-UC-3, T24, and EJ, were purchased from the Cell Bank of the Chinese Academy of Sciences (Shanghai, China). All the bladder cancer cell lines were cultured in an RPMI 1640 medium supplemented with 10% fetal bovine serum (FBS) and SV-HUC-1 was cultured in Ham’s F-12K medium with 10% FBS. All cell lines were maintained at 37°C in a 5% CO2 mammalian cell-culture incubator.

### The real-time quantitative PCR analysis

Human bladder tumor tissues and para-carcinoma tissues were collected and preserved from the patients experiencing radical cystectomy in Wuhan Union Hospital as described before (Zhang et al., 2021). This study was approved by the Ethics Committee of Wuhan Union Hospital of Huazhong University of Science and Technology (I2020I IEC-J (022). We collected 12 pairs of frozen bladder specimen and extracted the total RNA through a TRIZol reagent (Invitrogen, 15596026) and measured the total RNA by SYBR Green One-Step qRT-PCR kit (Invitrogen, 11736059). The specific details of the primers are shown in Supplementary Table S3. The relative expressions of these genes in normal and tumor tissues were presented in “PCRdata,” and the clinicopathological data of the 12 pairs of tissues were presented in the “Clinical pathological data for the tissues used for PCR”.

### Statistical analysis

DAVID (david.ncifcrf.gov) was used to perform Kyoto Encyclopedia of Genes and Genomes (KEGG) pathway analysis. GO analyses were conducted by using the clusterProfiler package of R software, and the online website Image GP (http://www.ehbio.com/ImageGP/) was used to display the results of the GO analyses. Spearman correlation analysis was used to conduct correlation analysis. Kruskal–Wallis and Wilcoxon tests were used for statistical tests. The “surv-cutpoint” function searched for the best separation cutoff value in survival analysis using the “survminer” R package. All statistical *p* values were two-sided, with *p* < 0.05 considered statistically significant. All data processing was performed in the R 4.0.3 software.

## Results

### The landscape of cuproptosis regulators in bladder cancer

**FIGURE 1:**
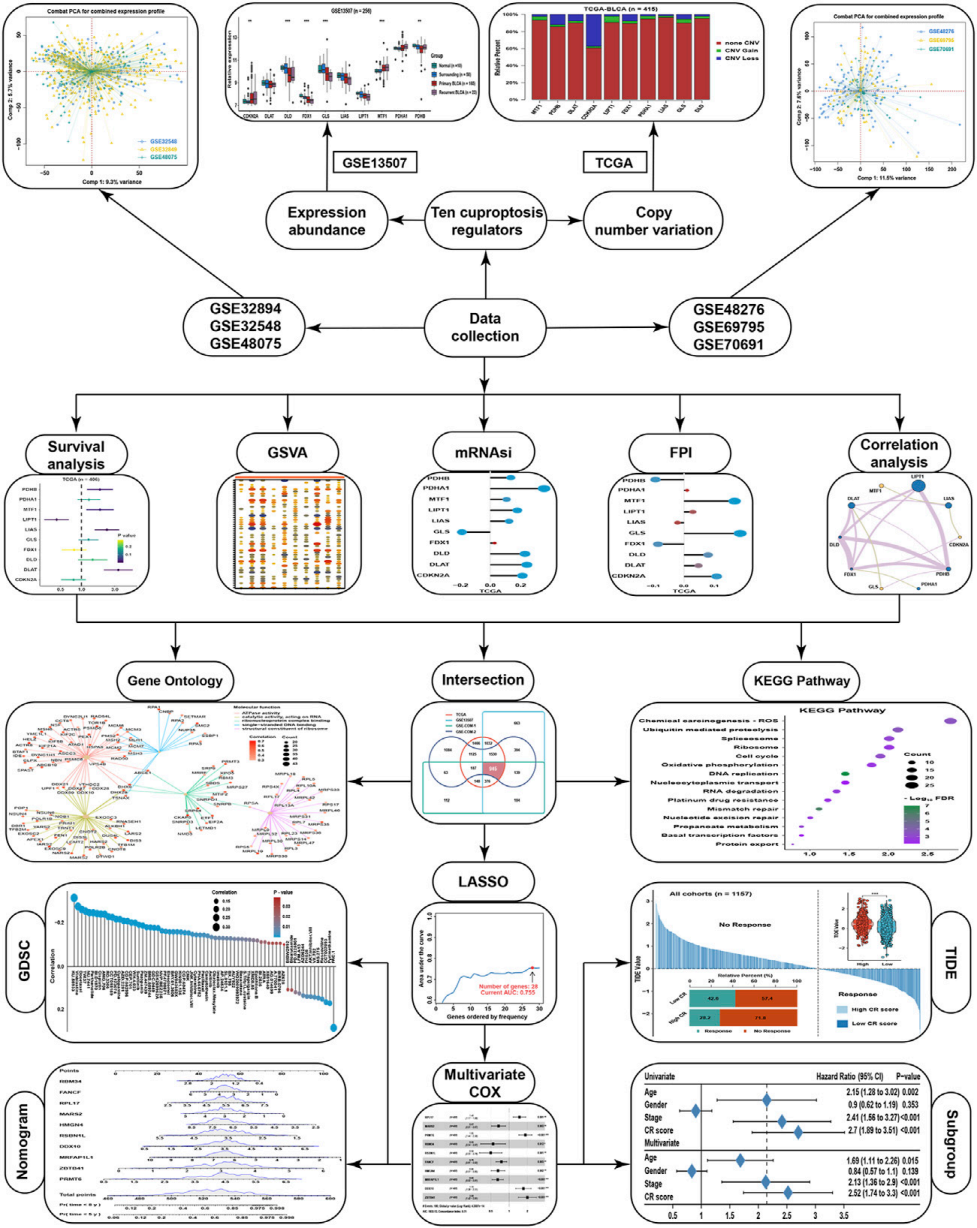
The flowchart of this study.

**FIGURE 2:**
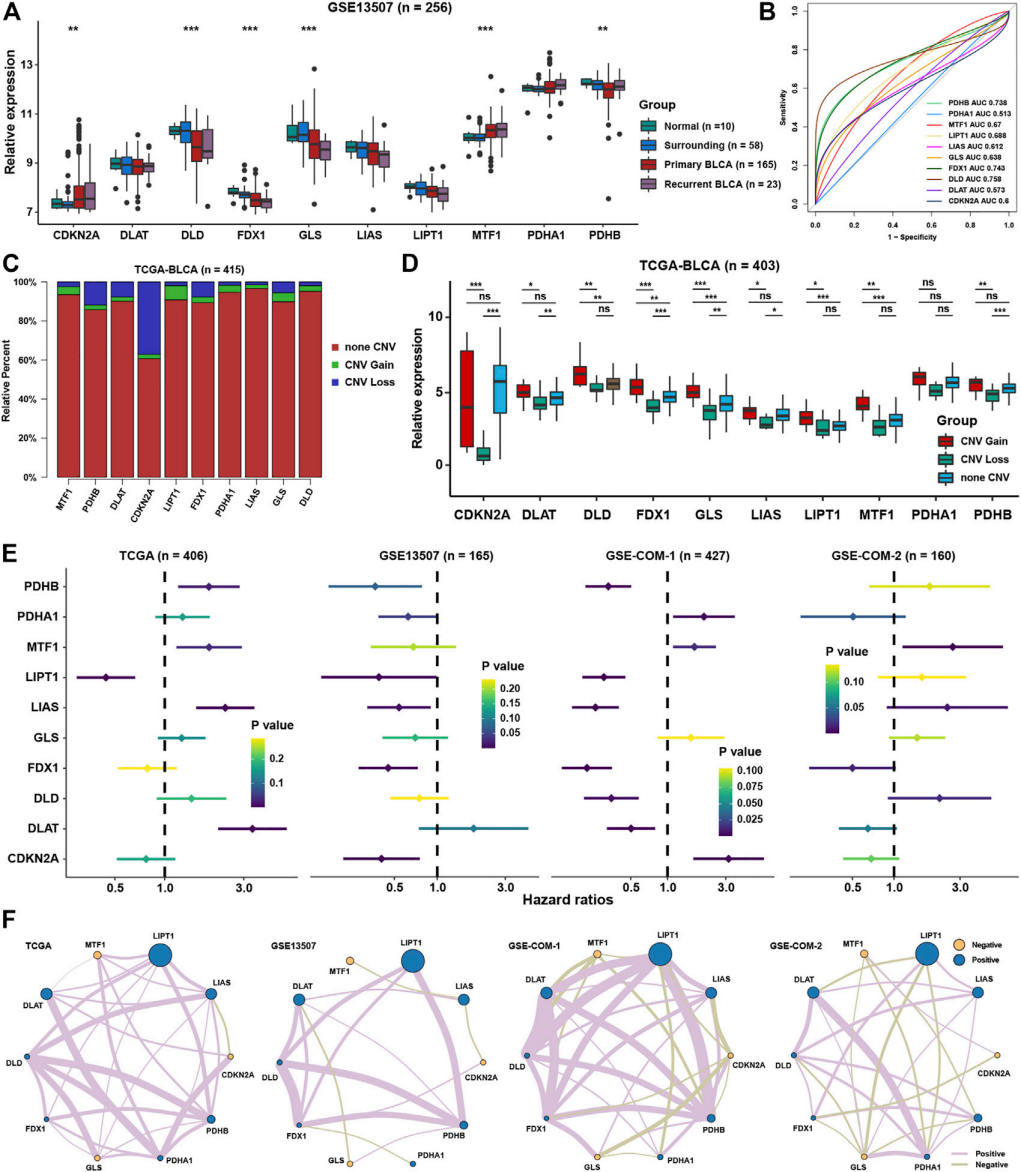
The landscape of cuproptosis regulators in BLCA. **(A)** Relative expression of ten cuproptosis regulators in different groups in BLCA. **(B)** AUC for ten cuproptosis regulators between tumor and normal tissues in BLCA. **(C)** Relative frequency percentage of copy number variation of ten cuproptosis regulators in the BLCA. **(D)** Relative expression of ten cuproptosis regulators in different CNV groups. **(E)** The prognostic analyses for ten cuproptosis regulators in four BLCA cohorts. **(F)** Correlation network among ten cuproptosis regulators in four BLCA cohorts. The thickness of the lines represented the strength of correlation. *P < 0.05, **P < 0.01, ***P < 0.001.

The workflow of this study is displayed in the flow chart (Figure 1). From a basic function perspective, differentially expressed analysis was important to explore whether the genes possessed research value. Currently, ten regulatory genes explicitly related to cuproptosis have been found (Tsvetkov et al., 2022), including 7 positive regulatory genes FDX1, LIAS, LIPT1, DLD, DLAT, PDHA1, and PDHB, and 3 negative regulatory genes MTF, GLS, and CDKN2A. In the GSE13507 cohort, we could find that the expression of CDKN2A and MTF1 were significantly higher in BLCA than in normal or surrounding tissues, while DLD, FDX1, GLS, LIAS, and PDHB were lower in BLCA than in normal or surrounding tissues (Figure 2A). As for diagnostic efficiency, ROC analysis was conducted for these cuproptosis regulators, and only PDHB, FDX1, and DLD displayed strong diagnostic power with AUC >0.7 (Figure 2B). In the TCGA cohort, we analyzed the relative percentage of CNV for cuproptosis regulators in BLCA, and combined with expression levels we found that almost all CNV losses of the cuproptosis regulators could account for the aberration of the expression levels (Figure 2C). Except PDHA1, the expressions of all cuproptosis regulators were lower in the CNV-loss group than in the CNV-gain group. The expressions of DLD, FDX1, GLS, LIPT1, and MTF1 were lower in a non-CNV group than in the CNV-gain group, while the expressions of CDKN2A, DLAT, FDX1, GLS, LIAS, and PDHB were higher in the non-CNV group than in the CNV-loss group (Figure 2D). Only FDX1 and GLS entirely satisfied the relationship between the change tendency of CNV and expression levels, which meant that the genetic variation might contribute to the function of them in the carcinogenesis of BLCA. To make the conclusion of the follow-up study more rigorous, we conducted normalization and batch removal of data from the same sequence platform in the GEO database so that these data could be combined into a larger dataset. Among them, GSE32548, GSE32894, and GSE48075 are combined as the GSE-COM-1 dataset (Supplementary Figure S1A), while GSE48276, GSE69795, and GSE70691 are combined as the GSE-COM-2 dataset (Supplementary Figure S1B). To investigate the prognostic value of cuproptosis regulators, survival analyses were conducted in four datasets (Figure 2E). Only MTF1, LIPT1, FDX1, and CDKN2A could be simultaneously satisfied with significantly prognostic values in at least three datasets. We further established an expression network among all cuproptosis regulators in four datasets by using correlation analysis, and we could find that only MTF1, GLS, and CDKN2A showed a negative relationship with other cuproptosis regulators in most of the datasets which was consistent with the basic function of positive or negative regulation in the process of cuproptosis (Figure 2F). It indicated that the regulatory relationship of cuproptosis regulators might indeed exist in BLCA.

### Functional characteristics of cuproptosis regulators in bladder cancer

**FIGURE 3:**
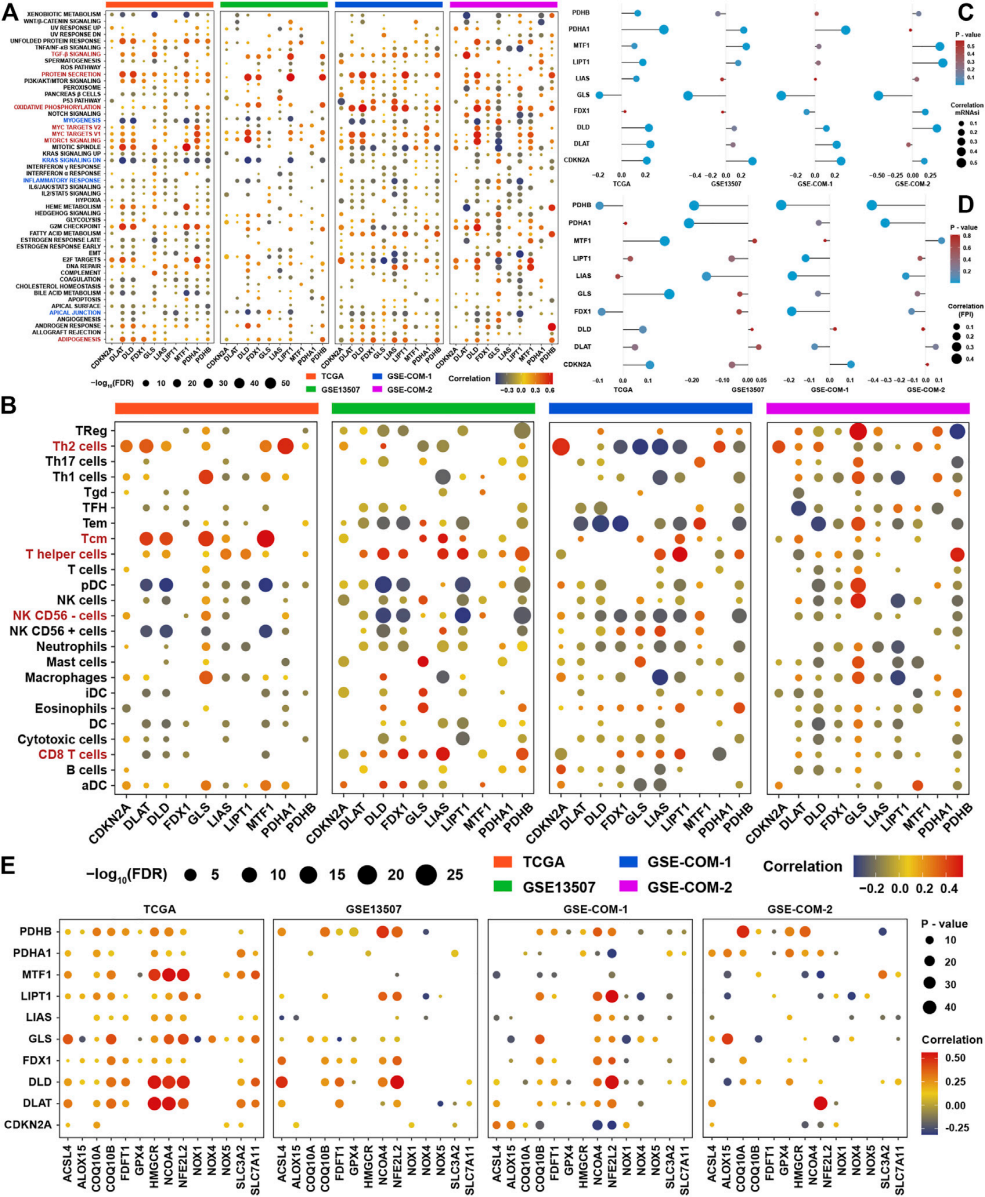
Functional characteristics of cuproptosis regulators in BLCA **(A)**. Correlation analysis among GSVA scores and the expression of ten cuproptosis regulators in the four BLCA cohorts **(B)**. Correlation analysis among infiltration levels of immune cells and the expression of ten cuproptosis regulators in four BLCA cohorts. Vacant positions represent no statistical significance between the term and cuproptosis regulator. The size of dots showed the correlation strength between regulators and function terms. The depth of the color indicates the strength of the correlation **(C)**. The correlation analysis between ten cuproptosis regulators and mRNAsi in the four BLCA cohorts **(D)**. The correlation analysis between ten cuproptosis regulators and the ferroptosis index in the four BLCA cohorts **(E)**. The correlation analysis among ten cuproptosis regulators and ferroptosis regulators in the four BLCA cohorts.

In view of novel insights into cuproptosis in recent days, systematically functional characteristics of cuproptosis regulators might be helpful in identifying their regulatory patterns in BLCA. The correlation results of the expression of cuproptosis regulators and GSVA enrichment scores revealed that most cuproptosis regulators showed inconsistent correlation tendency with GSVA enrichment scores in four datasets (Figure 3A). But several signaling pathways were highly positively associated with most cuproptosis positive regulators, such as TGF-β, protein secretion, oxidative Phosphorylation, MYC targets, MTORC1, and adipogenesis. At the same time, several signaling pathways were highly negatively correlated with cuproptosis positive regulators, such as myogenesis, KRAS, inflammatory response, and apical junction. From the results of the four datasets, we could simply find that most of the immune cells were correlated with cuproptosis regulators, such as CD8^+^ T cells, NK cells, and other types of T cells (Figure 3B). For further validation of the aforementioned results, we employed mRNAsi and FPI to explore the most promising cuproptosis regulators in regulating the metabolism and metastasis phenotypes in BLCA. We could find that GLS was the most relevant cuproptosis regulator, which was highly negatively correlated with mRNAsi, while CDKN2A was the most relevant cuproptosis regulator which was positively correlated with mRNAsi (Figure 3C). Due to same regulated by metal ions, we wanted to identify the relationship between ferroptosis and cuproptosis. Correlation analysis showed that PDHB was the most relevant cuproptosis regulator with a ferroptosis index, and PDHA1, LIAS, and FDX1 also showed correlation with FPI (Figure 3D). To further explore the relationship among ferroptosis and cuproptosis regulators, the correlation analysis showed that most of them showed high correlation with each other, especially for NCOA4 and NFE2L2 (Figure 3E). We speculated that there may be crosstalk between signaling pathways associated with cuproptosis and ferroptosis, because both were initiated by metal ions, and a strong correlation were found among the core regulators, which still need further experiment research.

### Identification and function prediction of cuproptosis-related genes

**FIGURE 4:**
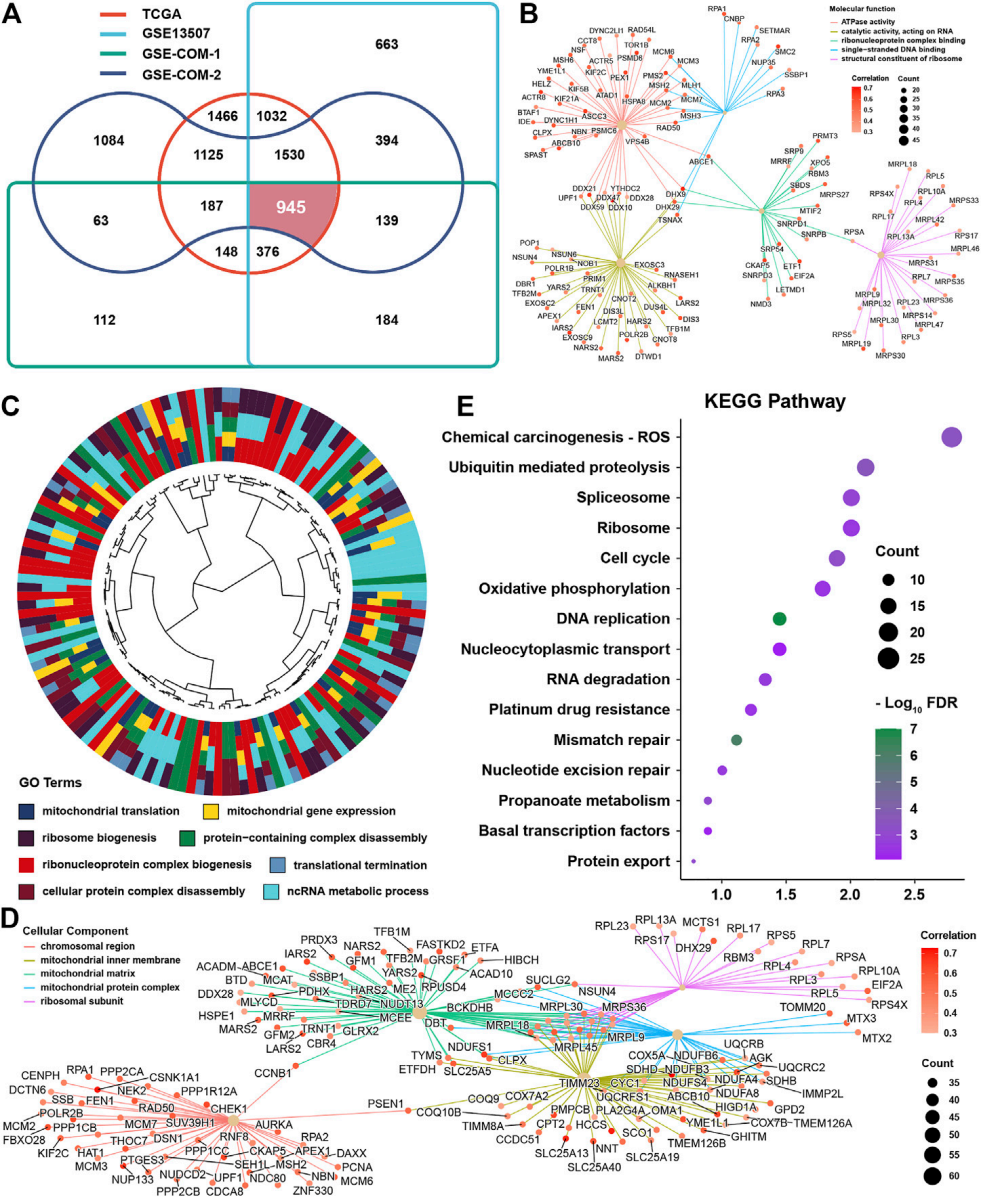
Identification and function prediction of cuproptosis-related genes **(A)** Intersection results from four datasets for cuproptosis-related genes **(B)**. Molecular function enrichment for cuproptosis-related genes **(C)**. Biological function enrichment for cuproptosis-related genes **(D)**. Cellular component enrichment for cuproptosis-related genes **(E)**. KEGG pathway enrichment for cuproptosis-related genes.

To investigate cuproptosis-related genes, correlation analyses were conducted in four independent datasets among cuproptosis regulators and messenger RNAs. After intersection of the results from four datasets, 945 genes were satisfied with correlation coefficients >0.3 and *p*-value <0.05 (Figure 4A). GO analysis was conducted for these genes, and we found that these genes were highly enriched in molecular function terms, such as ATPase activity, catalytic activity, acting on RNA, single-stranded DNA binding, ribonucleoprotein complex binding, and structural constituent of ribosomes (Figure 4B). As for biological function, we could find that the mainly enriched pathways were mitochondrial translation, mitochondrial gene expression, ribosome biogenesis, protein-containing complex disassembly, and ncRNA metabolic process (Figure 4C). Not surprisingly, these genes were mainly located in the mitochondrial inner membrane, matrix or protein complex, and ribosomal subunit (Figure 4D). KEGG analysis showed that these were enriched in ROS, ubiquitin-mediated proteolysis, spliceosome, ribosome, cell cycle, and oxidative phosphorylation (Figure 4E).

### Constructing a prognostic signature in bladder cancer

**FIGURE 5:**
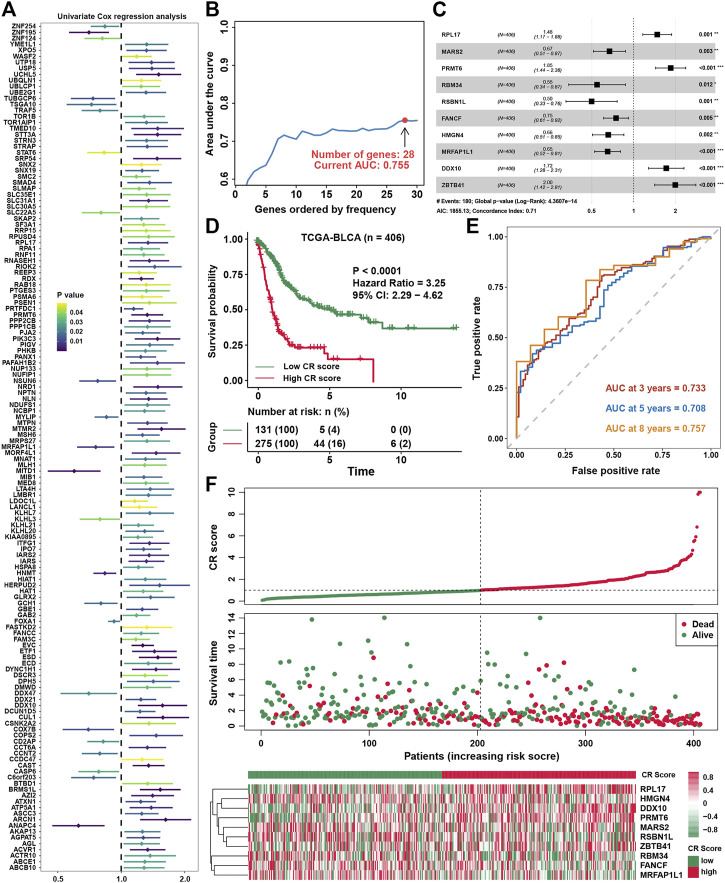
Constructing a prognostic signature in BLCA **(A)**. Univariate COX regression analysis for cuproptosis-related genes **(B)**. The variation tendency of AUC with the change of genes ordered by frequency **(C)**. Multivariate COX regression for constructing a prognostic signature in BLCA **(D)**.The survival analysis for low- and high-CR scores in BLCA **(E)**. ROC curves plotted for 3‐, 5‐, and 8‐y overall survival **(F)**. The vital status of patients in the high‐risk and low‐risk groups with changes in CR scores, and a heatmap of the expression profiles of members in the gene signature.

**FIGURE 6:**
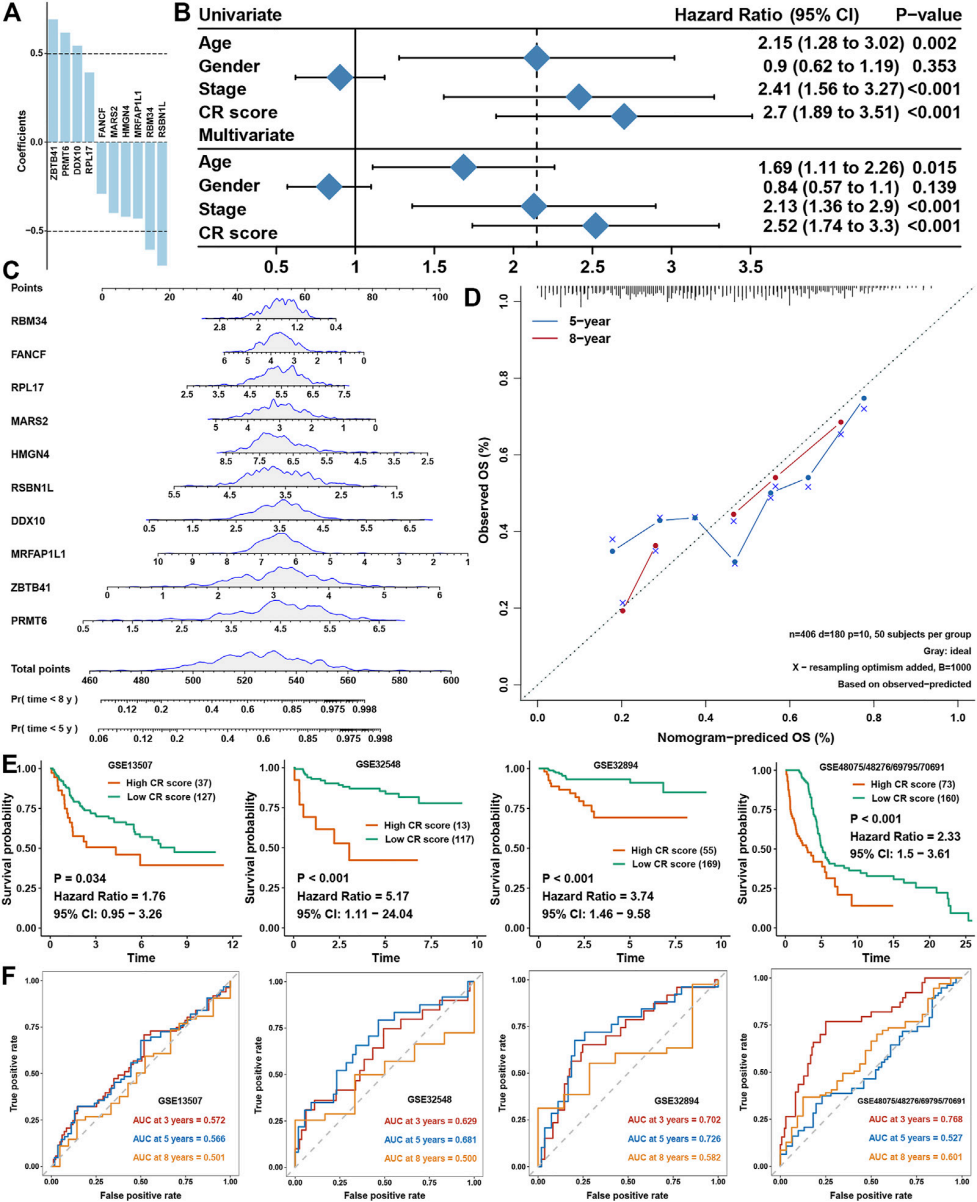
Validation of CR scores in BLCA **(A)**. Coefficients of members involved in the CR-scoring system **(B)**. Univariate and multivariate COX analyses for clinical traits and CR scores **(C)**. A nomogram to predict the 5‐y and 8‐y OS of BLCA **(D)**. The calibration curve for the nomogram model and the dashed diagonal line represents the ideal nomogram, the blue line and red line represent the 5‐y and 8‐y observed nomograms, respectively **(E)**. Validation of the CR scores in four external BLCA cohorts for overall survival **(F)**. ROC curves plotted for 3‐, 5,‐ and 8‐y OS of CR scores in the four external BLCA cohorts.

Univariate COX regression analysis showed that 141 genes were prognostic factors in BLCA (Figure 5A and Supplementary Table S3). To construct a perfect model, iteration LASSO was used to select feature genes that could be found in a high frequency in repetitious LASSO operations. After 1,000 iterations, 28 genes were selected for further analysis (Figure 5B). Multivariate COX regression analysis was used to construct a prognostic signature, and a ten-gene signature was set up with a concordance index of 0.71 (Figure 5C). The ultimate scores were called cuproptosis-related (CR) scores, and the CR scores were calculated using the following formula: CR scores = ZBTB41 * 0.692453 + PRMT6 * 0.617085 + DDX10 * 0.543223 + RPL17 * 0.3934 + FANCF * −0.29078 + MARS2 * -0.39922 + HMGN4 * −0.42016 + MRFAP1L1 * −0.43038 + RBM34 * −0.60586 + RSBN1L * −0.69634. The survival analysis showed that CR scores could predict the prognosis of BLCA patients well, the high CR score group showed a worse prognosis than the low CR score group (Figure 5D). ROC analysis showed that AUC at 3, 5, and 8 years were all higher than 0.7, which meant that the efficacy of the CR scores to predict survival was excellent (Figure 5E). With increase in the CR scores, the number of dead people was increasing, and the expression of ten members in the CR signature performed differences between the low and high CR score groups (Figure 5F). We could find that ZBTB41 and RSBN1L were the highest two genes in the aspect of coefficients in the signature (Figure 6A); meanwhile, they also had the most predictive ability of survival prognosis in BLCA. From univariate and multivariate COX analyses, we could find that age, stage, and CR scores were independent prognostic factors for BLCA (Figure 6B). For further clinical use, we established a nomogram for this CR score-calculating system (Figure 6C), and the calibration test showed that the nomogram-predicted survival rate displayed a high degree of consistency with the observed survival rate (Figure 6D). Moreover, we verified the predictive effect of CR signature in four validation datasets, and all datasets showed that the high CR score group had a worse prognosis than the low CR score group (Figure 6E). Eventually, ROC analyses were also conducted for CR scores in four validation cohorts to assess the 3-, 5-, and 8- year AUC, suggesting that CR scores could work well (Figure 6F).

### Functional characteristics of CR scores in bladder cancer

**FIGURE 7:**
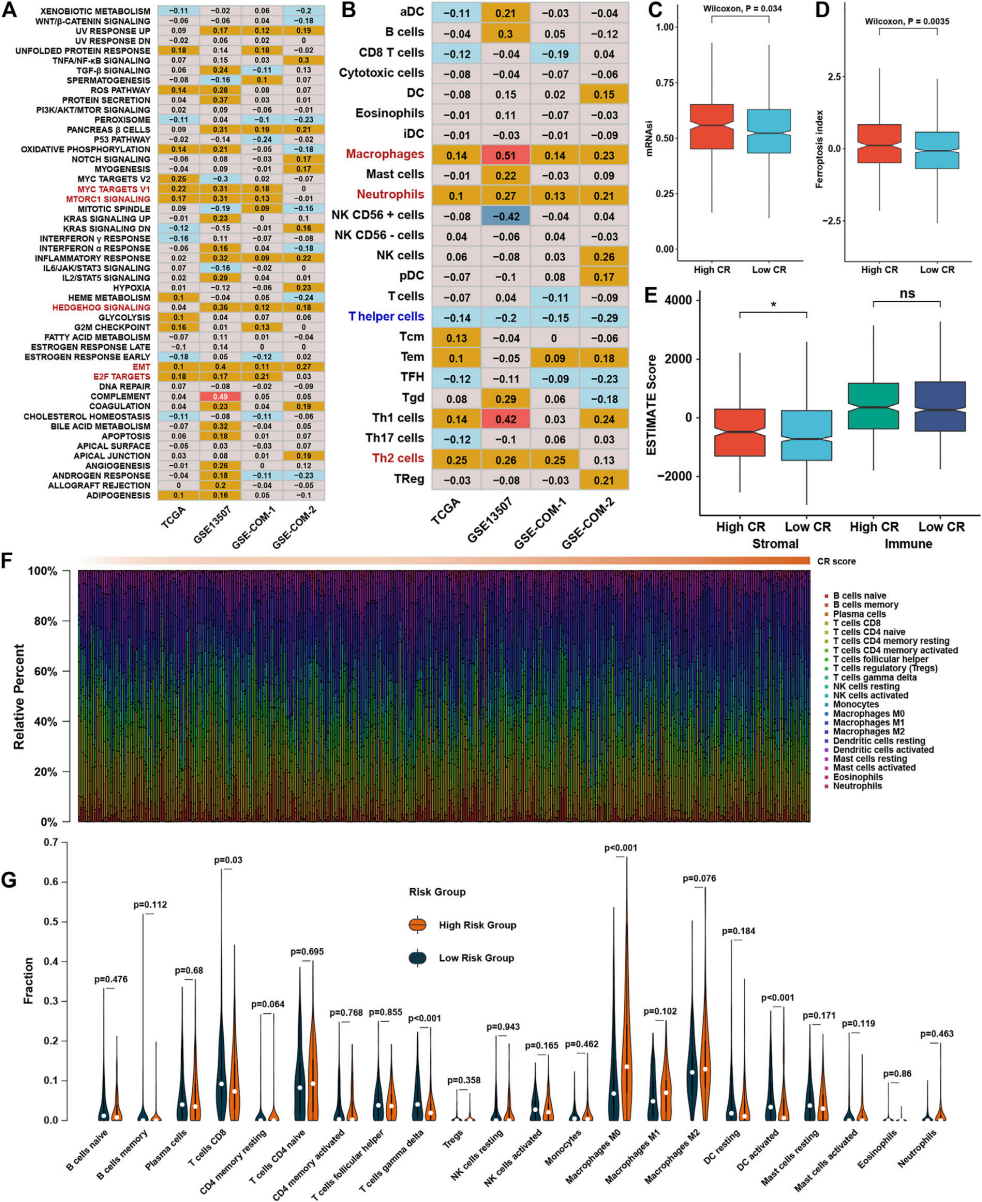
Functional characteristics of CR scores in BLCA **(A)**. Correlation analysis between CR scores and GSVA scores. The number represents the correlation coefficient of the two objects at the intersection **(B)**. Correlation analysis between CR scores and infiltration levels of immune cells **(C)**. The abundance of mRNAsi in different CR score groups **(D)**. The abundance of FPI in different CR score groups **(E)**. The abundance of ESTIMATE scores in different CR score groups **(F)**. The distribution of 22 immune cells calculated by CIBERSORT with change of CR scores, different colors represent different immune cell types **(G)**. Violin plot of the relative infiltration level of immune cells between different CR score groups.

Correlation analysis between GSVA enrichment scores and CR scores showed that CR scores was positively associated with EMT, MYC, MTORC1, HEDGEHOG, and E2F signaling in at least three datasets (Figure 7A). The correlation analysis between immune cell infiltration levels and CR scores showed that the CR scores were highly positively correlated with macrophages, neutrophils, and TH2 cells (Figure 7B). The high CR score group owned a higher mRNAsi and FPI than the low CR score group (Figures 7C,D). However, ESTIMATE showed that stromal scores was higher in the high CR score group than the low CR score group (Figure 7E), while there was no difference between low and high CR scores in immune scores. To be more rigorous, CIBERSORT was also conducted (Figure 7F), and we could find that the fraction of CD8^+^ T cells, γδT cells, and activated dendritic cells were significantly higher in the low CR scores group than in the high CR scores group, while macrophages were on the contrary (Figure 7G).

### Prediction of chemotherapy and immunotherapy efficacies of CR scores in bladder cancer

**FIGURE 8:**
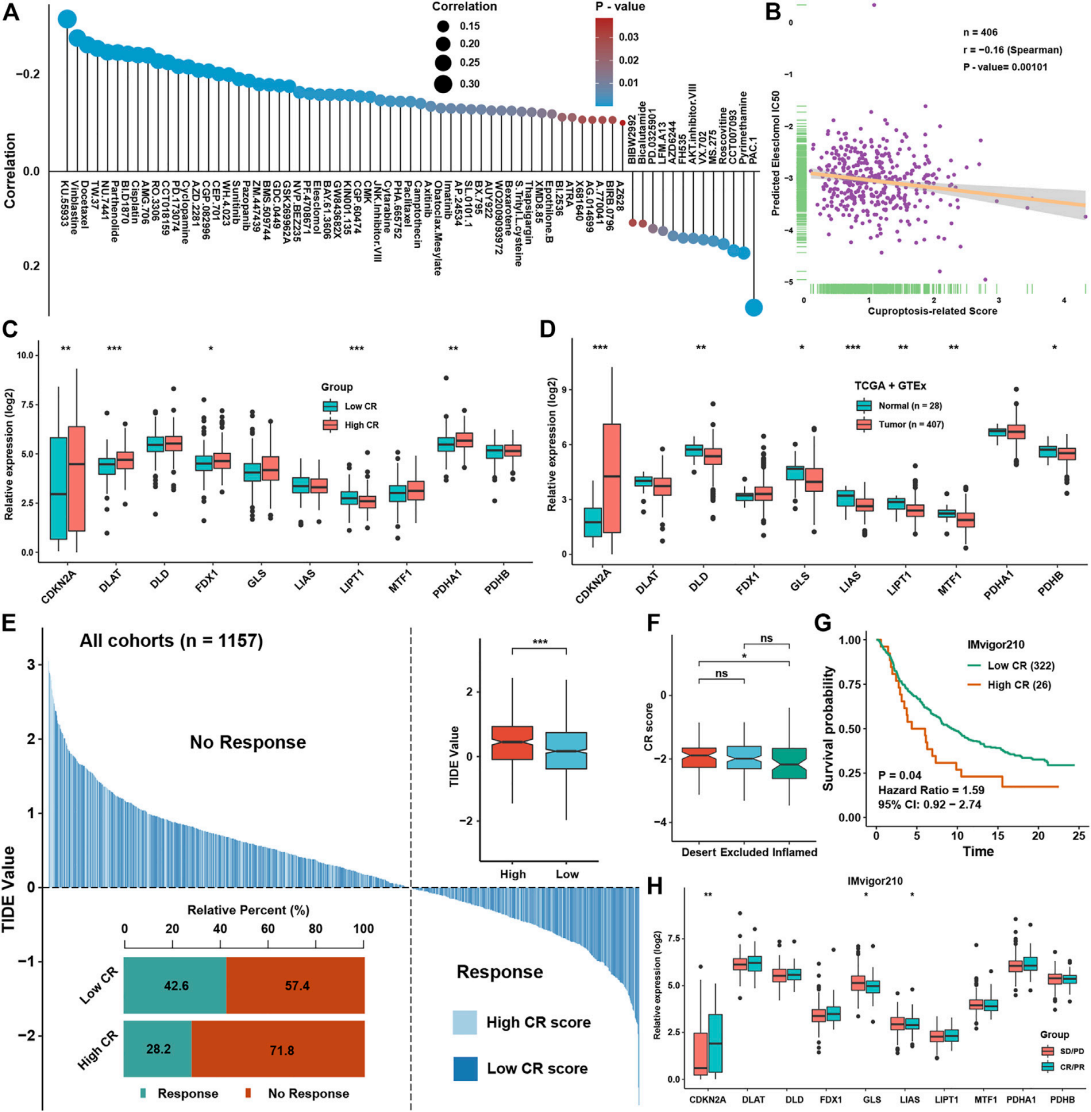
Prediction of chemotherapy and immunotherapy efficacies of CR scores in BLCA **(A)**. The correlation analysis between CR scores and prediction IC50 of drugs in the GDSC dataset **(B)**. The correlation analysis between CR scores and prediction IC50 of Elesclomol in the GDSC dataset **(C)**. The expression levels of cuproptosis regulators between low and high CR score groups **(D)**. The expression levels of cuproptosis regulators between normal and BLCA tissues in GTEx and TCGA datasets **(E)**. The TIDE value of BLCA samples was shown for different CR score groups, and the chi-square test used to assess significant differences is shown in the bottom-left side. The risk scores and TIDE values in different response groups and CR groups are shown in the upper-right side **(F)**. The abundance of CR scores in different immune subtypes in the IMvigor210 cohort **(G)**. Survival analysis for CR scores in the IMvigor210 cohort **(H)**. The abundance of ten cuproptosis regulators in different immune response groups in the IMvigor210 cohort. *P < 0.05, **P < 0.01, ***P < 0.001.

CR scores could predict the prognosis of BLCA patients well and was highly associated with some signaling pathways’ activation and infiltration levels of several immune cells, and we aimed to explore whether CR scores could predict the therapeutic efficacy in BLCA. Correlation analysis between IC50 of drugs in GDSC and CR scores showed that the low-CR group was more sensitive to the treatment of vinblastine, docetaxel, and cisplatin, which were frequently used in BLCA, while the high-CR group showed resistance to most chemotherapy drugs (Figure 8A). Especially, predicted IC50 of a cuproptosis inducer, elesclomol, was significantly negatively correlated with the CR score, indicating that inducing cuproptosis might be a potential therapeutic method for BLCA patients possessing higher CR scores (Figure 8B). We found that CDKN2A, DLAT, FDX1, and PDHA1 were significantly higher in the high-CR group, while LIPT1 was lower in the high-CR group, showing that most of the cuproptosis-positive regulators were higher in the CR score group (Figure 8C). We also found that most of cuproptosis-positive regulators including DLD, LIAS, LIPT1, MTF1, and PDHB were lower in BLCA tissues, while cuproptosis-negative regulator CDKN2A was significantly higher in BLCA tissues (Figure 8D). These indicated that elevating the levels of cuproptosis of BLCA patients might be a potential technology to improve treatment efficacy. Based on expression profiles, we predicted the efficacy of immunotherapy for BLCA patients in all cohorts by using TIDE analysis. We could see that the TIDE scores were lower in the low-CR group than in the high-CR group, which meant that the low-CR group had better efficacy in immunotherapy than the high-CR group (Figure 8E). To further validate the prediction power for the CR scores of immunotherapy efficacy, we selected the IMvigor210 cohort as an immune dataset for validation. We found that the CR scores was significantly higher in an immune-desert group than in the immune-inflamed group (Figure 8F) and was a risk factor for BLCA patients (Figure 8G). Moreover, the expression of CDKN2A was higher in the CR/PR group, while the expressions of GLS and LIAS were higher in the SD/PD group in the IMvigor210 cohort (Figure 8H).

### The validation of the expression of the genes of the signature

**FIGURE 9:**
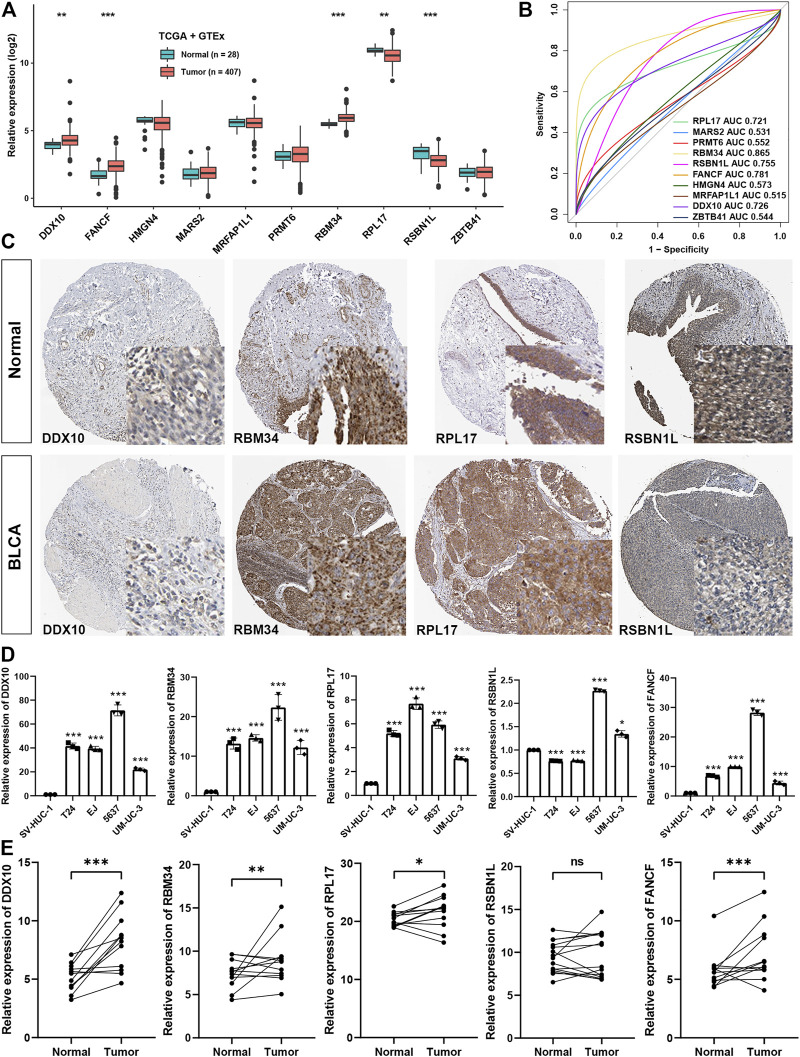
The validation of the expression of the genes of the signature **(A)**. The expression levels of genes involved in CR score signature between normal and BLCA tissues in GTEx and TCGA datasets **(B)**. AUC for ten CR score signature members between tumor and normal tissues in GTEx and TCGA datasets **(C)**. Representative image of four members involved in CR signature in HPA dataset **(D)**. Experimental verification of the expression levels of the genes of the signature between normal cell line and BLCA cell lines through qRT-PCR **(E)**. Experimental verification of the expression levels of the genes of the signature between normal and BLCA tissues through qRT-PCR.

To further elucidate the importance of genes involved in CR score signature, we found that DDX10, FANCF, and RBM34 were significantly higher in BLCA tissues, while RPL17 and RSBN1L were lower in BLCA tissues (Figure 9A). ROC analysis showed that only AUC of these five genes were higher than 0.7, meaning greater diagnostic efficiency between normal and BLCA tissues (Figure 9B). Using the Human Protein Atlas, we confirmed that the expression of DDX10, RBM34, and RPL17 were remarkably expressed higher in BLCA tissues, while RSBN1L was higher in normal tissues (Figure 9C). Moreover, using qRT-PCR, we showed that the expression level of DDX10, RBM34, RPL17, and FANCF were markedly up-regulated in BLCA cell lines in comparison with a normal cell line, but the expression level of RSBN1L was uncertain between BLCA and normal cell lines (Figure 9D). Importantly, we collected twelve paired normal bladder tissues and bladder cancer tissues, and the results demonstrated that the expression of DDX10, RBM34, RPL17, and FANCF were elevated in tumor tissues, but there was no significant difference in the expression of RSBN1L between normal and bladder cancer tissues (Figure 9E).

## Discussion

In this study, we systematically analyzed the landscape of cuproptosis regulators in BLCA in the aspect of expression levels and prognosis values and underlying relationships with biological functions, immune cell-infiltration levels, mRNAsi, and FPI. First, from the aspect of expression levels and copy number variation, we speculated that PDHB, FDX1, and DLD were significantly differentially expressed in BLCA with diagnostic efficacy because the AUCs of them were more than 0.7 to differentiate tumor and normal tissue. We also found that PDHB and DLD were differentially expressed in colorectal cancer, while FDX1 was distinctly expressed in lung adenocarcinoma. The prognosis aspect in four independent datasets in BLCA, MTF1, LIPT1, FDX1, and CDKN2A were satisfied with prognostic values in most of the datasets. In other tumors, we also found that these genes were closely related to the prognosis results of patients.

These cuproptosis-related molecules have been reported to be related to the AMPK/mTOR/ULK1 pathway, NF-kB pathway, and P13K/AKT/YAP pathway (Bu et al., 2020; Huang et al., 2021; Khouja et al., 2022). It could be seen from the results that TGF-β, protein, oxidative phosphorylation, MYC targets, MTORC1, and adipogenesis were closely related to cuproptosis and cuproptosis-related molecules (Lee et al., 2012; Chen et al., 2021; Deng et al., 2021; Ameh et al., 2022; Xia et al., 2022). Ferroptosis, autophagy, and apoptosis were also reported to be associated with NF-kB, Nrf2-HO-1, JAK-STAT, and mTOR pathways. Interestingly, cuproptosis was also a way of cell death, so the relationship between these pathways and cuproptosis indicated that traditional cell death might have crosstalk with cuproptosis by regulating these pathways’ activation. However, the specific regulating patterns with specific cuproptosis regulators still need further experimentation.

Since cuproptosis regulators were related to phenotypes such as metastasis, etc., mRNAsi was used to evaluate the genes most related to cell stemness among cuproptosis regulators, and it was found that GLS was the most associated regulator with mRNAsi (Li et al., 2019; Mukha et al., 2021). To explore the two most popular cell death modes induced by metal ions so far, the relationship between FPI and ferroptosis-regulated gene expression was verified. It was found that PDHB and LIAS had the most significant crosstalk with iron death. However, there have been no studies that have reported the specific relationship between these two factors, which would be a promise research direction in the future.

Ferroptosis, apoptosis, autophagy, and various other cell death modes have been demonstrated to be highly correlated with immune cell infiltration. Ferroptosis, autophagy, and apoptosis were also formerly reported to influence the effect of immune therapy. From the results of our analysis, cuproptosis regulators were also highly correlated with infiltration levels of immune cells, such as CD8^+^ T cells, NK cells, and dendritic cells. Previous studies have confirmed that these cells play a key role in immunotherapy and the regulation of the immune microenvironment, and the degree of infiltration of these cells could be affected by various cell death methods, especially ferroptosis, immunogenic cell death (ICD), apoptosis, etc. Our results showed that cuproptosis may also affect the immune microenvironment and even the efficacy of tumor immunotherapy and so on. Intermolecular regulation was ubiquitous in our lives. However, cuproptosis regulators proposed in the previous literature were limited and could not fully reveal such a phenomenon. Therefore, we conducted a correlation analysis to further explore the genes that may regulate cuproptosis-related genes or were regulated by cuproptosis-related genes. Through correlation analysis, we not only found 945 genes that were significantly related to cuproptosis regulators, but also through the univariate COX regression model, iteration LASSO and multivariate COX regression models, filtering, and screening, finally identified 10 genes that could be used to build a prognostic survival model, and might be involved in the regulation of cuproptosis.

Recently, more and more researchers have begun to focus on the role of cuproptosis to predict the prognosis or assessing the condition of immune cell infiltration levels. A novel cuproptosis-related prognostic gene signature has been constructed in clear-cell renal cell carcinoma and melanoma to predict the prognosis and validated to be associated with immune cell infiltration (Bian et al., 2022). Moreover, cuproptosis-related lncRNAs were also comprehensively analyzed and used to construct a prognostic model in hepatocellular carcinoma and soft-tissue sarcoma (Han et al., 2022; Zhang et al., 2022). Our study was the first to comprehensively analyze the cuproptosis regulators in bladder cancer and different from other methods of modeling, we employed iteration LASSO to make our model more robust. Importantly, several independent datasets were used to validate our conclusions.

Importantly, using data mining and qRT-PCR, we determined three genes involved in the CR-score signature, including DDX10, RBM34, and RPL17, that were higher expressed in BLCA than in normal tissues. Previous studies proved that DDX10 could promote proliferation or metastasis of tumor cells in lung cancer (Liu et al., 2021), colorectal cancer (Zhou et al., 2022), and ovarian cancer (Gai et al., 2016). However, its role in bladder cancer has not been reported yet. It has been reported that RPL17 could promote proliferation and stemness of colorectal cancer through ERK and NEK2/β-catenin signaling pathways (Ko et al., 2022). As for RBM34, no studies have been reported on its role in the progression of cancers. However, in our study, we validated that these three genes were significantly abnormal in BLCA tissues and showed great power for predicting the survival rate and therapeutic efficacy. But there still needs some mechanism experiments to explore the reason how these genes could influence the process of cuproptosis.

In conclusion, we systematically analyzed 10 cuproptosis regulators from the perspective of expression levels, prognostic values, and associated biological functions in BLCA. Based on the LASSO and COX algorithms, the CR scores calculated by ten cuproptosis-related genes could be helpful in the investigation of BLCA prognostic prediction and therapeutic efficacy.

## Data Availability

The original contributions presented in the study are included in the article/Supplementary Material further inquiries can be directed to the corresponding authors.
