# Supplementary material for: Construction of cuproptosis-related gene signature to predict the prognosis and immunotherapy efficacy of patients with bladder cancer through bioinformatics analysis and experimental validation
Source: Front Genet. 2022 Nov 23;13:1074981. doi: 10.3389/fgene.2022.1074981 (PMC9728801; doi:10.3389/fgene.2022.1074981)

**Supplementary Figure 1. Dara pre-processing before using and combined different datasets for a complete cohort in same sequencing platforms.**

(A) The raw PCA for combined expression profiles containing GSE32548, GSE32894, GSE48075 was shown in left plot, and the combat PCA for combined expression profiles was shown in right panel.

(B) The raw PCA for combined expression profiles containing GSE48276, GSE69795, GSE70691 was shown in left plot, and the combat PCA for combined expression profiles was shown in right panel.


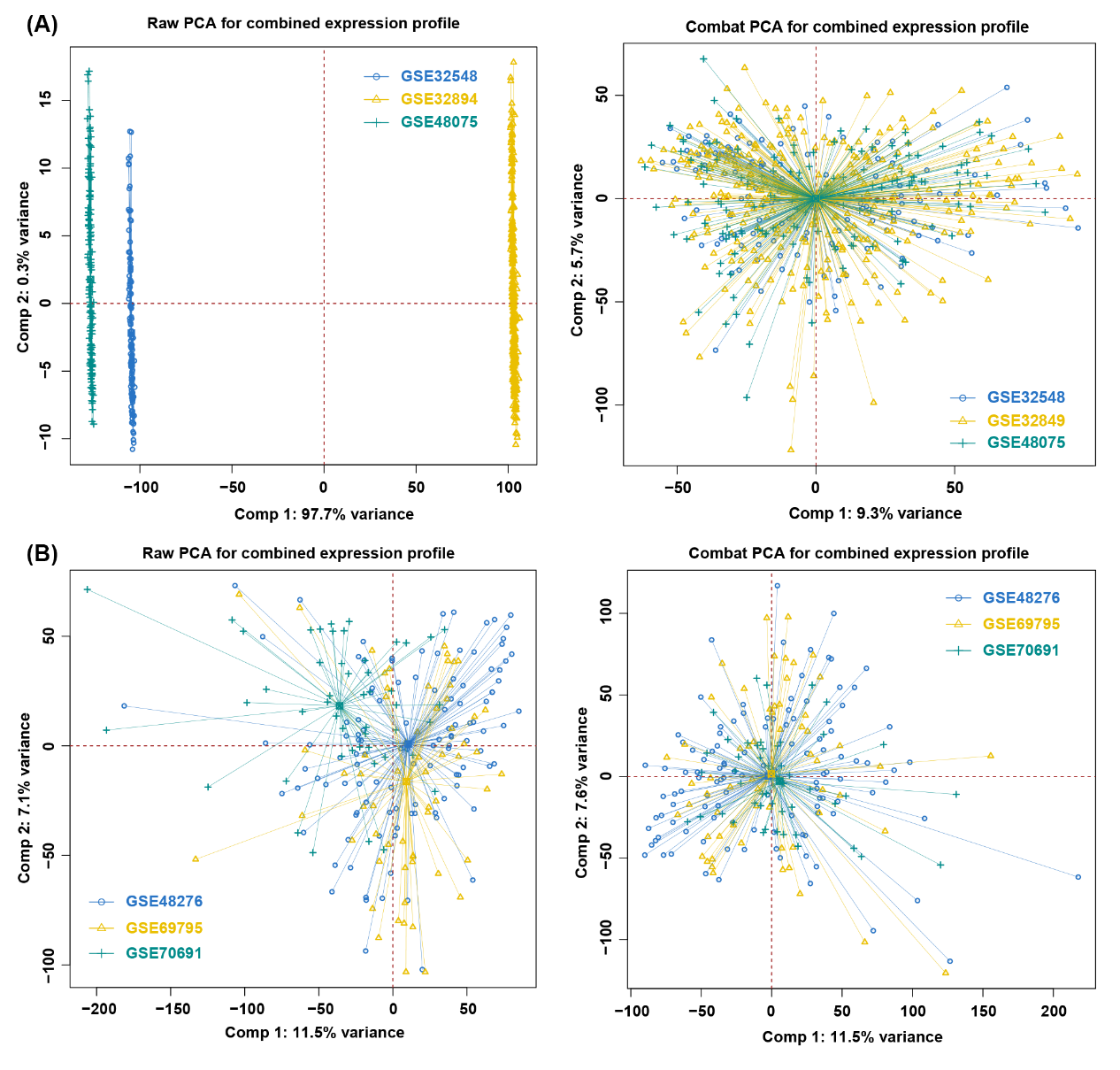

Supplement: Supplementary file 2 [file DataSheet1.docx]
